# A novel *CISD2* intragenic deletion, optic neuropathy and platelet aggregation defect in Wolfram syndrome type 2

**DOI:** 10.1186/1471-2350-15-88

**Published:** 2014-07-24

**Authors:** Enza Mozzillo, Maurizio Delvecchio, Massimo Carella, Elvira Grandone, Pietro Palumbo, Alessandro Salina, Concetta Aloi, Pietro Buono, Antonella Izzo, Giuseppe D’Annunzio, Gennaro Vecchione, Ada Orrico, Rita Genesio, Francesca Simonelli, Adriana Franzese

**Affiliations:** 1 Department of Translational Medical Science, Section of Pediatrics, Regional Center of Pediatric Diabetology, University of Naples “Federico II”, Via S. Pansini 5, 80131 Naples, Italy; 2 Pediatrics Unit, IRCCS Casa Sollievo della Sofferenza, San Giovanni Rotondo, FG, Italy; 3 Medical Genetics Unit, IRCCS Casa Sollievo della Sofferenza, San Giovanni Rotondo, FG, Italy; 4 Atherosclerosis and Thrombosis Unit, IRCCS Casa Sollievo della Sofferenza, San Giovanni Rotondo, FG, Italy; 5 Laboratory of Diabetology - Laboratory for the Study of Inborn Errors of Metabolism, Pediatric Clinic, Regional Center of Pediatric Diabetology, Istituto Giannina Gaslini, Genova, Italy; 6 Department of Molecular Medicine and Medical Biotechnology, University of Naples “Federico II”, Naples, Italy; 7 Multidisciplinary Department of Medical, Surgical and Dental Sciences, Eye Clinic, Second University of Naples, Naples, Italy

**Keywords:** CISD2, Optic neuropathy, Non-autoimmune diabetes mellitus, Novel mutation, Platelet aggregation, Sensorineural hearing loss, SNP-array, Upper intestinal ulcers, Wolfram syndrome

## Abstract

**Background:**

Wolfram Syndrome type 2 (WFS2) is considered a phenotypic and genotypic variant of WFS, whose minimal criteria for diagnosis are diabetes mellitus and optic atrophy. The disease gene for WFS2 is *CISD2*. The clinical phenotype of WFS2 differs from WFS1 for the absence of diabetes insipidus and psychiatric disorders, and for the presence of bleeding upper intestinal ulcers and defective platelet aggregation. After the first report of consanguineous Jordanian patients, no further cases of WFS2 have been reported worldwide. We describe the first Caucasian patient affected by WFS2.

**Case presentation:**

The proband was a 17 year-old girl. She presented diabetes mellitus, optic neuropathy, intestinal ulcers, sensorineural hearing loss, and defective platelet aggregation to ADP. Genetic testing showed a novel homozygous intragenic deletion of *CISD2* in the proband. Her brother and parents carried the heterozygous mutation and were apparently healthy, although they showed subclinical defective platelet aggregation. Long runs of homozygosity analysis from SNP-array data did not show any degree of parental relationship, but the microsatellite analysis confirmed the hypothesis of a common ancestor.

**Conclusion:**

Our patient does not show optic atrophy, one of the main diagnostic criteria for WFS, but optic neuropathy. Since the “asymptomatic” optic atrophy described in Jordanian patients is not completely supported, we could suppose that the ocular pathology in Jordanian patients was probably optic neuropathy and not optic atrophy. Therefore, as optic atrophy is required as main diagnostic criteria of WFS, it might be that the so-called WFS2 could not be a subtype of WFS. In addition, we found an impaired aggregation to ADP and not to collagen as previously reported, thus it is possible that different experimental conditions or inter-patient variability can explain different results in platelet aggregation. Further clinical reports are necessary to better define the clinical spectrum of this syndrome and to re-evaluate its classification.

## Background

Wolfram syndrome (WFS; MIM 222300) is a rare neurodegenerative disease with autosomal recessive inheritance and incomplete penetrance, whose diagnosis requires diabetes mellitus (DM) and optic atrophy (OA). WFS includes a wide spectrum of other possible disorders, such as diabetes insipidus, sensorineural deafness, genitourinary tract problems, male hypogonadism, neurological or psychiatric disorders, and less frequently, bowel dysfunction. Clinical wide spectrum is likely sustained by a genetic heterogeneity [1]. To date, 2 types of WFS, type 1 (WFS1; MIM 606201) and type 2 (WFS2; MIM 604928) characterized by different disease genes have been identified. The disease gene for WFS2 is *CISD2,* encoding for a highly conserved zinc-finger protein of the Endoplasmic Reticulum Intermembrane Small (ERIS), playing a pivotal role in calcium homeostasis (Ca2H) [2]. It was identified in three consanguineous Jordanian families carrying a point mutation in exon 2 [3]. In WFS2, diabetes insipidus and psychiatric disorders are not described, whereas bleeding upper intestinal ulcers (b-UIU), not reported in WFS1 [4], and defective platelet aggregation (PA) may be considered diagnostic criteria. After the first report [3,5], no further *CISD2* mutations have been reported. We describe the first Caucasian patient with WFS2 with a novel homozygous *CISD2* intragenic deletion.

## Case presentation

**Figure 1:**
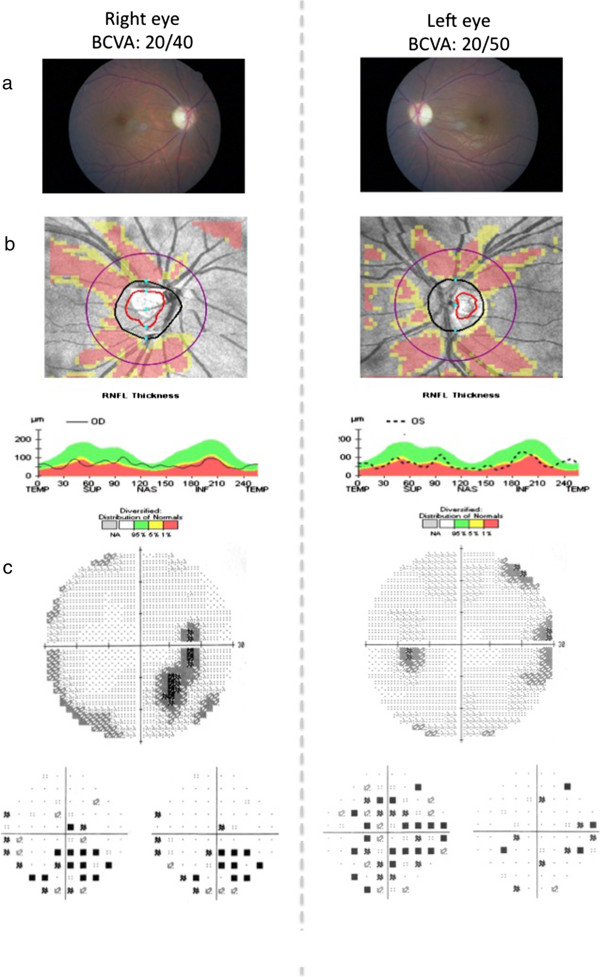
**The figure displays the results of ocular investigations. a)** Fundus ocular examination showing slight pallor of optic disc in both eyes, associated with best corrected visual acuity (BCVA) of 20/40 in the right eye and 20/50 in the left eye. **b)** Optical coherence tomography (OCT) retinal nerve fiber layer showing a moderate suffering of superior and inferior axonal fibers of optic nerve in both eyes and of nasal axonal fibers in the left eye. **c)** Visual field testing showing reduced retinal sensitivity in the temporal-inferior sector starting from the blind spot in the right eye and a generalized reduced retinal sensitivity reaching the nasal-inferior sector towards 30° in the left eye. The pattern visual evoked potentials (picture not shown) showed normal latency and normal amplitude responses in both eyes (latencies < 100 ms; amplitude of 6.2 μV in right eye and 5.9 μV in left eye [range of normal values 5.8 – 6.2 μV]).

Our patient was a Caucasian girl (FS), first child of non-consanguineous parents. Since the age of 5 years old, FS suffered from recurrent b-UIU. When 14 years old, malrotation and partial duodenum intussusception were diagnosed, resection of a duodenal tract and duodenal-jejunal anastomosis were performed. At the age of 16, she suffered from reduced visual acuity and dyschromatopsia, so she underwent eye examination including BCVA (20/40 in the right and 20/50 in the left eye), slit lamp biomicroscopy (normal anterior segment bilaterally), fundus examination (optic disc pallor with preserved pupillary light reflexes bilaterally), and p-VEP according to the ISCEV guidelines [6], (normal latency and normal amplitude responses bilaterally). The b-MRI showed normal optic nerve caliber in both eyes. At 17 years old non-autoimmune diabetes occurred and WFS2 was suspected. She started insulin treatment. Further examinations showed a bilateral sensorineural hearing loss in the high frequencies (SHLHF), and a slow intestinal transit time with entero-gastric bile reflux (EGBR). As visual acuity, pupillary light reflexes, slit lamp biomicroscopy, fundus examination and p-VEP were unchanged after 5 years follow up, additional eye exams were performed in order to extensively define optic nerve involvement. Visual field testing, ERG, MP, and OCT of macula and retinal nerve fiber layer showed a moderate involvement of the optic nerve in both eyes, compatible with a diagnosis of Optic Neuropathy (ON), excluding the OA (Figure 1) and other ocular abnormalities.

**Figure 2:**
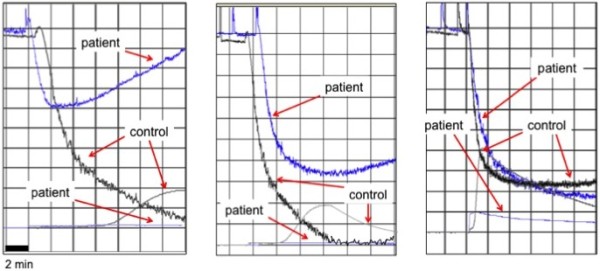
**The figure displays the platelet aggregation and secretion in response to ADP at different concentrations: 2 mM L**^**-1 **^**(left panel), 4 mM L**^**-1 **^**(central panel) 10 mM L**^**-1 **^**(right panel).** In the lower part of each figure, the wave corresponds to ATP secretion from platelets.

Evaluating PA, a reduced and reversible defective PA up to 10 μM of ADP was present, whereas PA in response to other tested agents (collagen, epinephrine, ristocetin) was within normal ranges. Platelet secretion in response to ADP was absent (Figure 2). The bleeding score, according to Tosetto et al. [7], was 3 (normal values: ≤3), the other main coagulative tests were normal.

Ophtalmological abnormalities, DM and deafness were not found in the parents and brother, whereas a reversible PA in response to ADP was found. Currently, FS is 22 years old and presents DM, ON with preserved visual acuity and electrophysiologic tests, no OA, tinnitus due to SHLHF triggered by loud environments, EGBR, slow intestinal transit, reduced and reversible defective PA to ADP, and b-UIU.

### Matherials and methods

DNA from the proband, parents, brother and controls were extracted from whole blood using High Pure PCR Template Preparation Kit (Roche, Mannheim, Germany). The three exons and the flanking region of CISD2 gene were amplified by PCR (PrimeStar GXL DNA Polymerase, Takara Bio Inc, Japan) using three couples of primers. Amplicons were purified and then sequenced for both sense and antisense strands. CISD2 cDNA sequencing was performed using known cDNA primers and conditions [3]. SNP-array analysis on trios was performed using the Affymetrix CytoScan HD Array (Affymetrix, Santa Clara, CA) as previously described [8].

Patients were enrolled after obtaining appropriate informed consent and approval by the local ethics committee (#08-CE) of the IRCCS Casa Sollievo della Sofferenza (San Giovanni Rotondo) where genetics studies were carried out.

### Identification of novel mutation in CISD2

In the patient the amplification of *CISD2* exon 2 was not detected, while exons 1 and 3 were regularly present. Moreover exons 1, 2 and 3 were amplified in parents, in brother and in controls. CISD2 cDNA sequencing revealed a novel homozygous deletion affecting the whole exon 2 of *CISD2* (see Additional file 1: Figure S1). To further confirm the exon 2 deletion, to map boundaries, and to confirm that her parents and brother were carriers, a SNP-array analysis on trios was performed. Log2Ratio values of markers encompassing the CISD2 deletion were of approximately -0.45 to -1, suggestive for a heterozygous deletion (mother, father and brother) while Log2Ratio values from -1 to higher negative values were suggestive for homozygous deletion.

**Figure 3:**
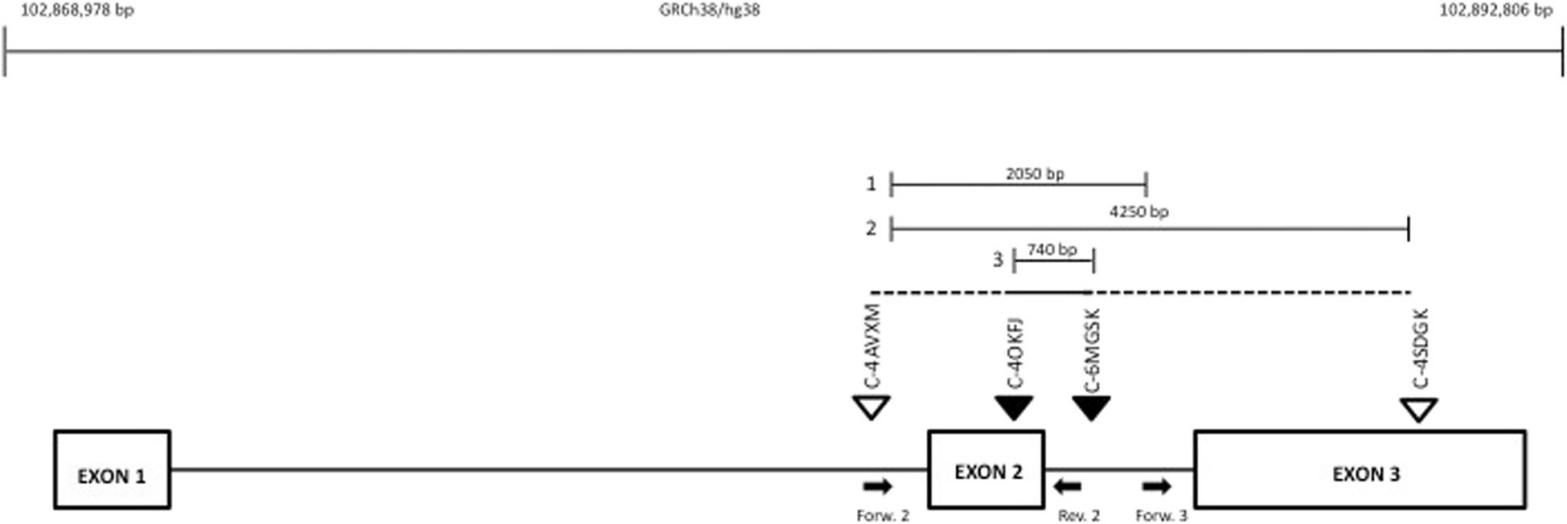
**Schematic representation of CISD2 gene and of the deleted region.** Black arrows represent PCR primers, unfilled triangles undeleted probes while filled triangles deleted probes. Line 1 represent the size of the deletion obtained by combining array and PCR data, line 2 and line 3 respectively the maximum size of the deletion and the minimum size of the deletion based on array results. On the upper line the coordinates of the region in hg38.

The analysis showed a deletion from C-4OKFJ (102,885,416 bp) to C-6MGSK (102,886,154 bp), which were the first and the last deleted oligonucleotide from the centromere, respectively (Table 1). The minimum size of deletion was 739 bp while the maximum size was 4,250 bp. Combining these data with the ones from PCR, we were able to map the proximal breakpoint (centromeric) between the last present probe C-4JSGI (102,885,132 bp) and the first deleted probe C-4OKFJ (102,885,416 bp), while the distal breakpoint (telomeric) between the last deleted probe C-6MGSK (102,886,154 bp) and the forward amplification oligo of CISD2 exon 3 (from 102,887,186 to 102,887,207 bp). Thus, the maximum deletion size has been restricted to a region of 2,050 bp spanning from intron 2 to intron 3. Base pair position were derived from the University of California Santa Cruz (UCSC) Genome Browser (http://genome.ucsc.edu/cgi-bin/hgGateway), build GRCh38 (Figure 3). The maximum deletion size was restricted to a region of 2,050 bp spanning from intron 2 to intron 3. The deletion was homozygous in the proband and heterozygous in her parents and brother. Since the mutation had never been reported, consanguinity was hypothesized. The patient’s parents reported to be not consanguineous even if their grandparents were born in the same Italian small island where they are still living. Long runs of homozygosity analysis from SNP-array data did not show any degree of parental relationship. The percentage of estimate consanguinity expressed in term of Coefficient of Inbreedeing was calculated as reported in Kearney et al. [9] (Table 2). Microsatellite analysis confirmed the hypothesis of a common ancestor (data not shown).

**Table 1:** Log2Ratio value of markers encompassing the CISD2 deletion on the CytoScan HD array

**Chromosome**	**Position (GRCh38)**	**Marker ID**	**Log2 Ratio**			
			**Patient**	**Brother**	**Father**	**Mother**
4	102,885,132	C-4JSGI	0.49391675	0.67028916	0.31938627	0.7485791
4	102,885,416	C-4OKFJ	-33.194.153	-0.6410765	-0.6913735	-0.86971235
4	102,885,416	C-3IWPX	-32.682.135	-0.6565456	-0.35076097	-0.6521124
4	102,885,641	C-6VJTI	-43.055.053	-10.099.052	-0.98227024	-17.431.074
4	102,886,154	C-6MGSK	-1.749.246	-0.5463655	-12.236.409	-0.75301945
4	102,889,381	C-4SDGK	-0.023144266	-0.23841621	0.05294265	0.06327062

**Table 2:** **The percentage of estimate consanguinity expressed in term of Coefficient of Inbreedeing and Identical by Descent**[9]

	**Coefficient of inbreeding**	**IBD (Identical by Descent %)**
Patient	0.0086908	0.86
Brother	0.002465	0.24

## Discussion

We describe the first European Caucasian girl with WFS2, carrying a novel intragenic exon 2 *CISD2* homozygous deletion. Only a homozygous mutation of the *CISD2*, a single nucleotide substitution that leads to a missense change Glu37Gln, was previously associated to WFS2 [3]. This gene, mapping on chromosome 4q24 [1], encodes an extremely small zinc-finger protein, named ERIS, which displays a fairly wide cellular expression profile, including pancreas, brain, blood and platelets [3]. Conlan et al. [10] showed that the domain 2Fe-2S is essential for the activity of this zinc-finger protein, and is located from aminoacid 99 (Cys) to aminoacid 112 (Gly). Genomic data from our subjects show that the triplets coding for aminoacids WRSKT, from the position 102 to 106 of the polypeptide, are within the deleted region, and this may result in a non functional protein because of zinc-finger domain lost. The ERIS protein is highly conserved and has 70% identity and 82% similarity across all the vertebrate species [3]. It plays a pivotal role in Ca2H [2], as well as wolframin, another endoplasmic reticulum (ER) [7] protein encoded by WFS1 gene. Also wolframin is involved in the regulation of ER stress and Ca2H [11,12], without any interaction with ERIS. The ER function influences some crucial secretory proteins, such as insulin, as well as cell-surface receptors and integral membrane proteins. Imbalance in Ca2H of ER elicits stress in this organelle, leading to the accumulation of misfolded and unfolded protein in the organelle, a state called ‘ER stress’, which plays, in WFS, a key role in β-cells, leaving these cells more prone to oxidative stress and consequently to apoptosis. Activation of the unfolded protein response (UPR), an adaptive response that counteracts the ER stress, serves to restore ER homeostasis. Moreover, UPR maintains pancreatic β-cell function and promotes β-cell survival. Exaggerated ER stress, which normally triggers the UPR, has been hypothesized in the β-cells impairing the cell progression and increasing apoptosis [13]. Neuronal cells have highly developed ER, whose "stress" is related with ganglion cells apoptosis, as demonstrated in a glaucoma chronic injury model [14]. The degeneration of ganglion and glial retinal cells could be related to the ER stress probably accountable for the ocular involvement in our patient that presented moderate ON but not OA. Few and non-specific studies on ERIS localization and expression, in particular in the ganglion and glial cells of the optic nerve, may explain the different optic nerve involvement and a favourable outcome of ocular pathology. In WFS1, early OA causes alterations of the optic nerve caliber due to the loss of a considerable number of ganglion cells [15]. These patients show severe progressive visual acuity reduction and non recordable p-VEP or increased latency or greatly reduced amplitudes [16,17]. In Jordanian WFS2 patients OA was observed after childhood or was described as “asymptomatic”, but opportune investigations were not reported [1]. Since our patient does not show OA at the age of 22 years, and since “asymptomatic” OA described in Jordanians is not completely supported, we suppose that the ocular pathology in those patients was probably ON and not OA. This is just a case report and thus great caution is needed, even if the absence of OA, one of the main diagnostic criteria of WFS, suggests that the so-called WFS2 could not be a WFS subtype.

As shown in Table 3 the clinical spectrum of WFS1 and WFS2 is only partially coincident, according to the different expression of the two proteins. In fact wolframin is expressed in pancreas, brain, heart, skeletal muscle, placenta, lung, liver and kidney, while ERIS is expressed in pancreas, brain, blood and platelets. Furthermore in WFS2 patients, diabetes insipidus and psychiatric disorders are not described so far, otherwise some additional features are present and may be considered diagnostic criteria: significant bleeding tendency, defective PA with collagen [4], and peptic ulcer disease with b-UIU [5]. The tendency to mucocutaneous bleedings mainly displayed by gastric ulcers is a common feature, observed in 79% and 90% respectively of Jordanian patients [5]. Expression studies using mass spectrometry listed in the Human Protein Reference Database (accession number 17413) show the presence of ZCD2 transcripts in platelets [3]. This may explain, at least in part, the bleeding phenotype [18] described in these patients.

**Table 3:** Clinical features of WFS1, WFS2, and of our case

***Clinical features***	***WFS 1***	***WFS2***	***WFS2 our case***
*Diabetes mellitus*	X	X	X
*Optic atrophy*	X	X	
*Sensorineuronal Deafness*	X	X	X
*Diabetes Insipidus*	X		
*Neurological and Psychiatric disorders*	X		
*Genitourinary tract problems*	X		
*Male hypogonadism*	X		
*Bowel disfunction*	X		X
*Upper intestinal ulcers*		X	X
*Platelet aggregation defects to collagen*		X	
*Platelet aggregation defects to ADP*			X
*Optic neuropathy*			X

Furthermore, in Table 3 we show the difference in the clinical spectrum of Jordanian cases [1] and our patient. Firstly, our girl presented ON and not OA even though as previously discussed it is possible that the ocular pathology in those patients was probably ON and not OA; secondly, she showed an impaired platelet aggregation to ADP and not to collagen; however collagen- and ADP- induced aggregation are both calcium-mediated, thus it is possible that different experimental conditions or inter-patient variability can explain different results in PA.

The new findings of this report could better characterize the WFS2 phenotype.

## Conclusion

We describe the first Caucasian patient with so-called WFS2. She carried a novel mutation in *CISD2*. Our data debate the previous description of WFS2 pointing out new data about eye and platelets involvement. The proband presented optic neuropathy and not optic atrophy, impaired platelet aggregation to ADP and not to collagen. Further clinical reports are necessary to better define the clinical spectrum of this syndrome and to re-evaluate its classification.

## Consent

This study was approved by institutional ethics committee and was done in accordance with Declaration of Helsinki. Informed consent for genetic analysis was obtained from the study participants. Moreover, as the patient is a girl, written informed consent was obtained from her parents for publication of this Case report. A copy of the written consent is available for review by the Editor of this journal.

## Abbreviations

WFS: Wolfram syndrome; DM: Diabetes mellitus; OA: Optic atrophy; WFS1: WFS type 1; WFS2: WFS type 2; ERIS: Endoplasmic reticulum intermembrane small; Ca2H: Calcium homeostasis; BCVA: Best corrected visual acuity; p-VEP: Pattern visual evoked potentials; ERG: Full-field electroretinogram; MP: Microperimetry; OCT: Optical coherence tomography; b-MRI: brain Magnetic Resonance Imaging; PA: Platelet aggregation; b-UIU: Bleeding upper intestinal ulcers; SHLHF: Sensorineural hearing loss in the high frequencies; EGBR: Entero-gastric bile reflux; ON: Optic neuropathy; ER: Endoplasmic reticulum; UPR: Unfolded protein response.

## Competing interest

The authors declare that they have no competing interests.

## Authors’ contributions

EM and MD made the clinical diagnosis and wrote the paper. GD and PB contributed to the diagnosis. AO and FS performed the ophtalmological examinations, wrote the ophtalmological section and provided the ophtalmological figures. FS reviewed and discussed the ophtalmological section. EG and GV performed the hemocoagulation testing, provided the platelet aggregation figure, wrote and discussed the hemocoagulation section. AS, CA and AI performed the molecular diagnosis. MC and PP confirmed the deletion and mapped boundaries through SNP-array analysis, performed the inbreeding through microsatellite analysis, provided tables and figure (on line suppl), wrote and discussed the molecular diagnosis section. RG supervised the entire molecular diagnosis. EM and MD edited the manuscript. AF supervised the manuscript. AF is the guarantor of this work and, as such, had full access to all the data in the study and takes responsibility for the integrity of the data and the accuracy of the data analysis. All authors read and approved the final manuscript.

## Pre-publication history

The pre-publication history for this paper can be accessed here:

http://www.biomedcentral.com/1471-2350/15/88/prepub

## Supplementary Material

Additional file 1: Figure S1 The upper part of the figure displays the PCR amplification of *CISD2* exons: agarose electrophoresis shows no exon 2 detection in proband (p) DNA. The lower part displays *CISD2* cDNA PCR amplification: agarose electrophoresis shows absence of proband (p) *CISD2* cDNA. From the left to the right: f: father, m: mother, p: proband, b: brother, ct+ ct-: controls, MW: molecular weight. Click here for file
