# Supplementary figures and images for: A novel CISD2 intragenic deletion, optic neuropathy and platelet aggregation defect in Wolfram syndrome type 2
Source: BMC Med Genet. 2014 Jul 24;15:88. doi: 10.1186/1471-2350-15-88 (PMC4121299; doi:10.1186/1471-2350-15-88)

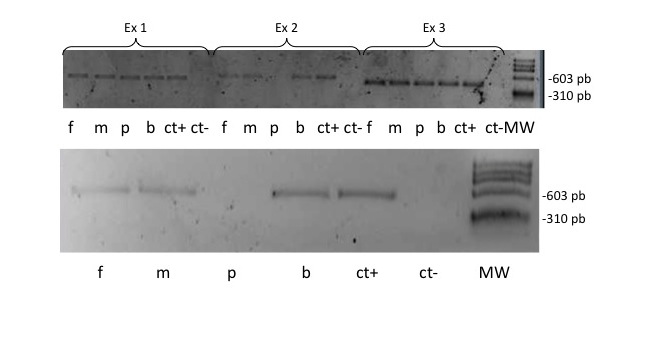

Supplement: Additional file 1: Figure S1 — The upper part of the figure displays the PCR amplification of CISD2 exons: agarose electrophoresis shows no exon 2 detection in proband (p) DNA. The lower part displays CISD2 cDNA PCR amplification: agarose electrophoresis shows absence of proband (p) CISD2 cDNA. From the left to the right: f: father, m: mother, p: proband, b: brother, ct+ ct-: controls, MW: molecular weight. [file 1471-2350-15-88-S1.jpeg]
